# Ethynylation of Formaldehyde over CuO/SiO_2_ Catalysts Modified by Mg Species: Effects of the Existential States of Mg Species

**DOI:** 10.3390/nano9081137

**Published:** 2019-08-07

**Authors:** Zhipeng Wang, Lijun Ban, Pingfan Meng, Haitao Li, Yongxiang Zhao

**Affiliations:** Engineering Research Center of Ministry of Education for Fine Chemicals, School of Chemistry and Chemical Engineering, Shanxi University, Taiyuan 030006, China

**Keywords:** Si-O-Mg structures, synergistic effect, Mg species, Reppe process

## Abstract

The highly effective catalytic synthesis of 1,4-butynediol (BD) from the Reppe process is a fascinating technology in modern chemical industry. In this work, we reported the effects of the existential states of Mg species in the CuO/silica-magnesia catalysts for the ethynylation of formaldehyde in a simulative slurry reactor. The physichemical properties of the supports and the corresponding catalysts were extensively characterized by various techniques. The experimental results indicated that the introduced Mg species in the form of MgO particles, MgO microcrystals, or Si-O-Mg structures effectively resulted in an abundance of medium-strong basic sites, which can synergize with the active Cu^+^ species, facilitate the activation of acetylene, and improve the ethynylation activity. For the CuO/MgO-SiO_2_ catalyst, the existence of Si-O-Mg structures strengthened the Cu–support interaction, which were beneficial to improving the dispersion and the valence stability of the active Cu^+^ species. The highly dispersed Cu^+^ species, its stable valence state, and the abundant medium-strong basic sites enhanced the synergistic effect significantly, leading to the superior activity and stability of CuO/MgO-SiO_2_. The insights into the role of the existential states of Mg species and the revelation of the synergistic effect between active Cu^+^ species and basic sites can provide theoretic guidance for future rational design of catalysts for the ethynylation reation.

## 1. Introduction

1,4-butynediol (BD), containing -C≡C- and -OH groups, is an important C4 feedstock with wide application in in electroplate, leatheroid, medicine and pesticide, etc. [1,2,3]. More significantly, through the hydrogenation of the unsaturated -C≡C-, BD can be industrially used to produce 1,4-butanediol (BDO), which serves as a crucial building block for synthesis of high-value added chemicals, such as 3-butene-1-alcohol (BTO), tetrahydrofuran (THF), polytetramethylene ether glycol (PTMEG), γ-butyrolactone (GBL) etc. [4,5,6,7,8,9,10]. Furthermore, the halogenated derivatives of BD with active reactivity can be pervasively used as synthetic intermediates in medicine, pesticides, spices and polymers [11]. The tremendous demand of BD and BD-derived products spur the increasing academic and industrial interests to develop cost-effective catalytic process for BD production.

The current industrial production of BD is via Reppe process—the catalytic ethynylation of formaldehyde over SiO_2_ supported CuO-based catalysts. It is generally believed that these catalysts are not active per se, rather the reaction of formaldehyde with acetylene is actually catalyzed by active Cu^+^ species (cuprous acetylide) formed in situ during the reaction process [3,11,12,13]. The transition of the Cu-based precursor into the active phase involves complex physicochemical changes that include the reduction of Cu^2+^ to Cu^+^ in the formaldehyde/acetylene atmosphere and the carbonization of Cu^+^ with acetylene [14,15]. However, during the reaction, the active Cu^+^ species are prone to spontaneous aggregation and over-reduction to metallic Cu particles, which decrease the catalytic performance drastically [11,12]. For the conventional CuO/SiO_2_ catalytic system, the weak Cu-SiO_2_ interaction can always give rise to the migration and the irreversible aggregation of active phase and the formation of inactive metallic copper, resulting in the severe deactivation in the ethynylation reaction [13]. Therefore, to strengthen the Cu-support interaction and stabilize the Cu^+^ species become a challenging task for designing and developing high-performance CuO/SiO_2_ catalysts over ethynylation reaction.

In the open literature, many efforts have been made to improve the activity and stability of CuO/SiO_2_ catalytic system in the ethynylation reaction [16,17,18,19,20,21,22,23]. It is reported in patents that adding bismuth oxide in the Cu/SiO_2_ catalyst can effectively inhibit the transformation of Cu^+^ to Cu^0^ and thus extend the life-span of the catalysts [11]. Besides the Bi_2_O_3_, Hort et.al [24] found that introduction of Mg species into the CuO-Bi_2_O_3_/SiO_2_ catalyst can further enhance the activity in the ethynylation reaction. The reason is possibly due to the enhancing role of Mg species in the Cu-support interaction, originating from the chemical interaction between the copper and the lattice of the silica-magnesia supports, which is just a simple speculation and lacks the direct evidence for detailed characterization of the catalysts. In spite of the unclear promotion mechanism of Mg species, more and more researchers have gradually approved the catalytic systems containing Cu, Bi, Si, and Mg elements, which has been employed in the industrial production. Moreover, in recent years, our groups, Zheng and the coworkers, prepared various highly efficient CuO-Bi_2_O_3_/SiO_2_-MgO catalysts via different methodologies, but they still did not specify the role of Mg species [25]. It is implied that Mg species plays a pivotal role in the ethynylation of formaldehyde, nevertheless, the promotion mechanism of Mg species remains ambiguous and need to be further investigated.

As a typical kind of alkali earth compounds, MgO are featured with various base sites on the surface, such as strong (low coordination O^2−^ anions), medium (oxygen in Mg^2+^-O^2−^ pairs) and weak (OH- groups) basicity [26,27,28]. Except for the intrinsic basicity, the addition of Mg can exert a crucial impact on the surface acid-base property of the SiO_2_ support, which relys heavily on the existence forms of Mg species [29,30,31]. Huang et al. [29] and Carlo Angelici et.al [30] prepared a series of binary MgO-SiO_2_ materials with different recombination between MgO and SiO_2_ via various method. They demonstrated that the existence forms of Mg species, especially Si-O-Mg bonds, dominates the balance of surface acid/base sites. Furthermore, numerous studies have shown that the acid-base property of the support can cause strong Cu-support interaction, thus inhibiting the agglomeration and growth of copper specise particles, thereby improving the stability of the catalyst [32,33]. Although some studies have focused on significant importance of Mg species in tuning the surface property and the interaction of copper-support, very little work, as far as we know, have been carried out to unfold the vital role of Mg species with different forms in the ethynylation reaction and establish the structure-activity relationships of the catalysts. 

In this paper, CuO/silica-magnesia catalysts with different Mg species were employed for the ethynylation of formaldehyde under atmospheric pressure. For comparison, the CuO/SiO_2_ was also prepared by deposion-precipitation method and used for the same reaction. The silica-magnesia supports with different Mg species and the corresponding catalysts were well characterised by XRD, N_2_ adsorption-desorption, H_2_-temperature programmed reduction (H_2_-TPR), CO_2_-temperature programmed desorption (CO_2_-TPD) and X-ray photoelectron spectroscopy (XPS) etc. techniques to correlate the ethynylation performance with their physicochemical properties.

## 2. Materials and Methods

### 2.1. Catalyst Preparation

SiO_2_-MgO support was prepared referring to our previous work. Firstly, the Tetraethyloxysilane (TEOS) (15 mmol) was dissolved in anhydrous ethanol (60 mL) followed by the addition of water (15 mL). Then, Mg(NO_3_)_2_ (5 mmol) was dissolved in 60 mL of anhydrous ethanol and 15 mL of water, which was added to the above mixed solution. And the solution was stirred at 333 K for 12 h. Subsequently, the aged gel was washed with anhydrous ethanol to remove water contained in the gel. Finally, the gel was dried in a stainless tank via ethanol super critical fluid drying (SCFD). According to the above processs, the SiO_2_ aerogel was prepared in the same way. By means of the incipient wetness impregnation method, MgO/SiO_2_ samples with the same Mg content were prepared. All the samples (MgO-SiO_2_, MgO/SiO_2_, SiO_2_) were calcined at 723 K in air for 3 h.

With CuCl_2_ as copper source, Cu (20 wt%) supported catalysts were prepared by ultrasound-assisted deposion-precipitation method. First, 2.265 g CuCl_2_ was completely dissolved in 150 mL of deionized water, and then 2.4 g SiO_2_ or silica-magnesia support and 50 mL of polyethylene glycol-400 were added to form a suspension. And 150 mL NaOH (0.1 M) was added dropwise to the suspension under ultrasonic stirring at 333 K. The mixture was filtered off, and then washed with deionized water, dried at 363 K overnight, followed by a calcination in air at 723 K for 3 h. The prepared catalysts were denoted as Cu/MgO-SiO_2_, Cu/MgO/SiO_2_, and Cu/SiO_2_, respectively.

### 2.2. Characterization Methods

The phase structure of the supports and the catalysts were determined by Powder X-ray diffraction (XRD) pattern using Bruker D8 Advance diffraction spectrometer with a target of Cu Kα (λ = 0.15418 nm).

The contents of metal elements were determined by inductively coupled plasma atomic emission spectroscopy (ICP-AES, Perkin Elmer Optima 7300 DV).

N_2_-physisorption analysis was measured on a Micromeritics ASAP-2020 apparatus. Before the adsorption analysis, the fresh and used samples were degassed at 150 °C for 5 h and 60 °C for 24 h, respectively. Specific surface area was obtained from the 5-point Brunauer–Emmett–Teller (BET) procedure. The average pore diameter and pore volume were determined by the Barrett-Joyner-Halenda (BJH) method.

Temperature-programmed desorption (TPD) of NH_3_ or CO_2_ were acquired on the same apparatus. The samples (100 mg, 40–60 mesh) were pretreated at 500 °C under a flow of helium (99.999%) at a rate of 60 mL min^−1^ for 60 min and saturated with a flow of pure NH_3_ or CO_2_ after cooling to 100 °C. These pretreated samples were then purged in a helium atmosphere at 100 °C until the baseline was stable in order to remove physisorbed NH_3_ or CO_2_, after which these samples were heated from 50 °C to 600 °C at 10 °C min^−1^ under a flow of helium. The amount of NH_3_ or CO_2_ evolved from the sample was determined using a thermal conductivity detector (TCD).

Fourier-transform infrared (FT-IR) spectra were recorded on a Bruker Tensor 27 Fourier transform infrared spectrometer at room temperature by subtraction of the background reference. One milligram of each powder sample was diluted with 100 mg of vacuum-dried IR-grade KBr. 16 scans were collected for each sample at a resolution of 4 cm^−1^.

Pyridine-infrared (Py-IR) spectroscopy was performed using an in situ vacuum adsorption infrared characterization system at the Dalian Institute of Chemical Physics. Self-supporting tablets samples (20 mg) were placed in an in-situ reaction cell, pretreated under vacuum at 120 °C for 90 min, allowed to adsorb pyridine at room temperature, treated under vacuum at 100 °C for 30 min, and allowed to return to room temperature, after which the IR spectrum was recorded using a Bruker Tensor 27 Fourier-transform infrared spectrometer.

CO adsorbed infrared spectra (CO-IR) of the used catalysts were recorded on a Bruker Tensor 27 Fourier transform infrared spectrometer. The used samples were evacuated at 60 °C for 3 h to remove the impurities on the surface. After cooling to room temperature, the in-situ diffraction reflectance reaction cell was filled by a controlled stream of CO-Ar (10% CO) at a rate of 5.0 mL·min^−1^ for 30 min. After the saturated adsorption, the determination were carried out.

Diffuse reflectance ultraviolet visible (DRS UV-Vis) spectra were obtained under ambient conditions on a CARY 300 ConcVarian spectrophotometer with BaSO_4_ as the standard reference material. 

X-ray photoelectron spectroscopic (XPS) and Auger electron spectroscopy (XAES) were performed in an ultra-high vacuum chamber (PHI 1257) with a base pressure of −4 × 10^−10^ Torr. The XPS spectrometer was equipped with a high resolution hemispherical electron analyzer (279.4 mm diameter with 25 meV resolution) and a Mg (Kα) (*hν* = 1253.6 eV) X-ray excitation source. All the spectra were referenced to adventitious C (1s) at 284.5 eV binding energy (BE).

Transmission electron microscopy (TEM) was carried out using a Jeol JEM-2100 transmission electron microscope operated at 200 kV. The diameters of more than 200 randomly selected metal particles were measured and the corresponding metal particle size distributions (PSDs) were determined. Based on these PSDs, the average particle diameter (d) was calculated according to the expression *d* = ∑*nidi*/∑*ni*, where ∑*ni* > 200.

### 2.3. Evaluating Catalytic Activity

0.5 g prepared catalyst and 10 mL of aqueous formaldehyde (28 vol%) were added into a 100-mL round bottom three-port flask equipped with a thermometer and a condenser tube. N2 gas was injected to exhaust the air in the flask. Meanwhile, the temperature of the reaction system was raised to the prescribed temperature. Turning on stirring, introducing C2H2 gas, and turning off N2 gas to start the reaction. After 15 h of reaction, the used catalyst was seperated from the reaction mixture, washed alternately by deionized water and ethanol, and stored in vacuum oven under 30 °C. The content of BD in the reaction solution was determined by Agilent 7890A gas chromatograph fitted with a DB-5 capillary column (0.32 mm × 50 m) and an FID detector. The unconverted formaldehyde in solution was determined by iodimetry [11,12].

## 3. Results

### 3.1. Characterization of the Supports

#### 3.1.1. Structure and Texture Properties of the Supports

**Figure 1:**
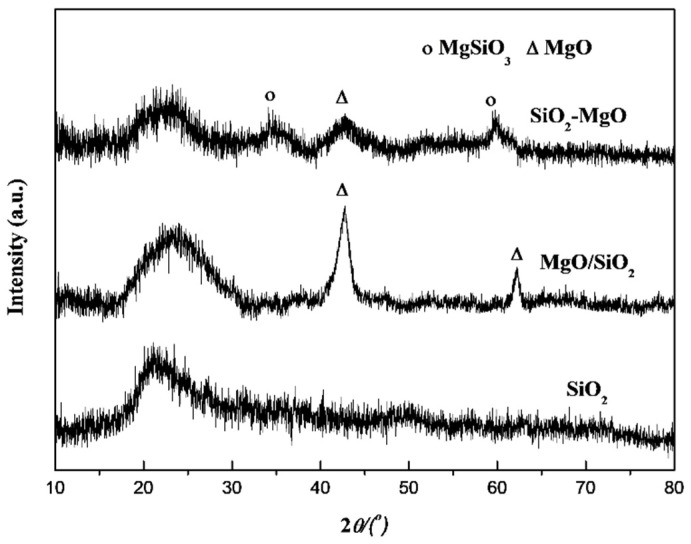
XRD patterns of the supports.

Figure 1 shows the XRD patterns the supports with clear different bulk phase property. For all the samples, the broad diffraction peaks at about 22° were attributed to amorphous silica [34]. Besides the characteristic diffraction of SiO_2_, for MgO/SiO_2_ samples, the diffraction peaks at 42.7° and 62.2° can be well attributed to the (200) and (220) faces of poplycrystalline MgO species [35]. Whereas, there was only one broad peak at 42.7° owing to MgO in SiO_2_-MgO samples. Compared with MgO/SiO_2_, the intensity and width of MgO (200) diffraction peak became much weaker and wider, suggesting MgO species in the SiO_2_-MgO have lower crystallinity, which was due to the higher dispersion of MgO species on the SiO_2_-MgO than that on MgO/SiO_2_. However, apart from the characteristic diffractions of MgO microcrystals, two peaks were observed at 35° and 60° for SiO_2_-MgO samples, which were ascribable to magnesium-silicate-hydrate species (M-S-H) [36,37].

**Figure 2:**
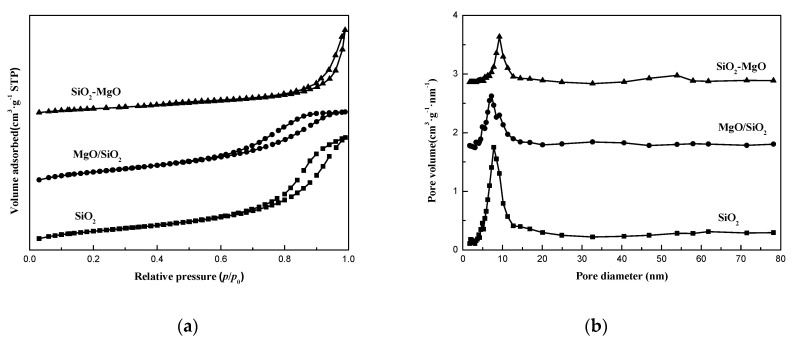
N_2_ adsorption-desorption isotherms (**a**) and pore size distributions (**b**) of the supports.

So as to investigate the evolution process of the supports texture property, the N_2_-physisorption analysis of the supports was measured. Figure 2 displayed the N_2_-adsorption/desorption isotherms and pore size distributions of SiO2 and silica-magnesia supports. In the light of the International Union of Pure and Applied Chemistry (IUPAC) recommendation, all the supports showed typical type IV isotherms with distinct hysteresis loops between *p/p*_0_ = 0.5–1.0 (Figure 2a), demonstrating that structures of the samples featured mesoporous frameworks with facile pore connectivity. However, great differences in the loops shape and *p/p*_0_ position were observed for the supports, indicating their different texture property. For the SiO_2_ and MgO/SiO_2_ supports, the adsorption/desorption isotherms showed the typical H2 hysteresis loops, which were caused by the accumulation of the nanoparticles. While SiO_2_-MgO exhibited an H3 shaped hysteresis loop, demonstrating that the pore structure of SiO_2_-MgO support is mainly characterized by slit-like pores formed by accumulation of aggregated particles. 

The pore size distribution was determined by using the Barrett-Joyner-Halenda (BJH) method from the desorption branch of the isotherms. As shown in Figure 2b, uniform mesopores around 9.1 nm for SiO_2_ were observed. The smaller pores around 8.8 nm for MgO/SiO_2_ reveals the blocking of the uniform mesopore structure of SiO_2_ aerogel by the incorporation of MgO. In contrast, SiO_2_-MgO sample possessed the double pore size distribution peak at about 11.0 and 56.2 nm, respectively, suggesting the collapse of mesopore structure of SiO_2_ aerogel and the formation of the bimodal porosity. As reported by Shin-Ichiro Fujita et al. [38], the different combination way between Si species and Mg species of silica-magnesia composites could affect the growth of mesopore channels.

The specific surface area and pore volume of the supports are summarized in Table 1, which clearly presented that the surface area of the supports in descending order followed: SiO_2_ > MgO/SiO_2_ > SiO_2_-MgO. The pore volume and average diameter of SiO_2_ are 2.31 cm^3^·g^−1^ and 9.44 nm, which are larger than those of MgO/SiO_2_ samples. Whereas, for SiO_2_-MgO samples, the pore volume decreased to 1.97 cm^3^·g^−1^ and average pore diameter increased to 10.05 nm since its special mesoporous framework.

#### 3.1.2. Identifying the Existential States of Mg Species

**Figure 3:**
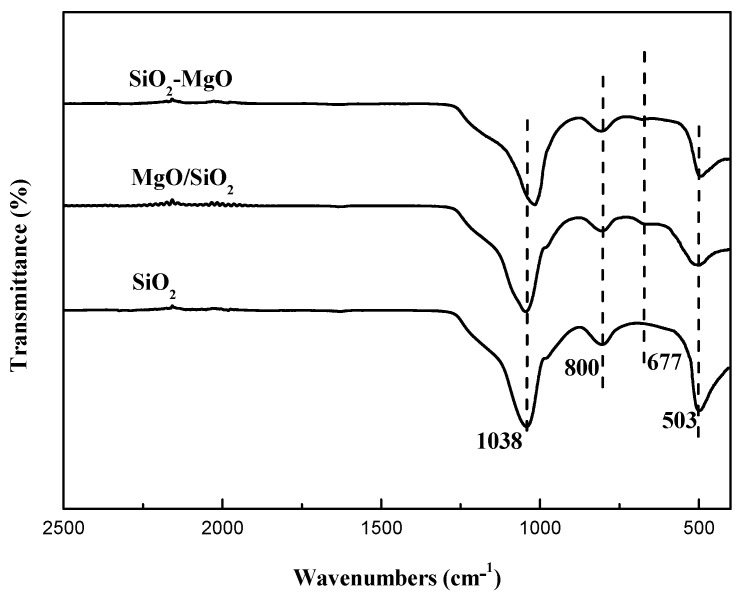
FT-IR spectra of the supports.

For the purpose of investigating the structural features of the magnesia–silica samples and assessing the nature of the interactions of SiO_2_ with MgO, the supports were determined by the FT-IR transmission spectra. Figure 3 presented the FT-IR spectra of the supports in the region of 4000–400 cm^−1^. The typical bands of SiO_2_ were similar to that reported in the literature, namely at 800 cm^−1^ originating from the symmetric stretching vibration (Si-O-Si), at 1038 cm^−1^ from the asymmetric stretching vibration (Si-O-Si), and at 503 cm^−1^ from the Si-O-Si bending mode [39,40]. The IR bands of MgO/SiO_2_ was similar to that of SiO_2_ except the Mg–O stretching vibration at 680 cm^−1^ attributed to MgO particles [41], which was in accord with XRD results and further proved MgO disperses on the surface of SiO_2_. For the SiO_2_-MgO samples, the symmetric stretching vibration Si–O–Si bands at 1038 cm^−1^ slightly shifted to a lower wave number. As reported by Huang et al. [29], the downshift of Si–O–Si bands has been observed by introduction of magnesium species, which was mainly induced by the formation of Si-O-Mg hetero-linkages. In addition, the weak band of Mg–O stretching vibration also observed in the SiO_2_-MgO samples.

**Figure 4:**
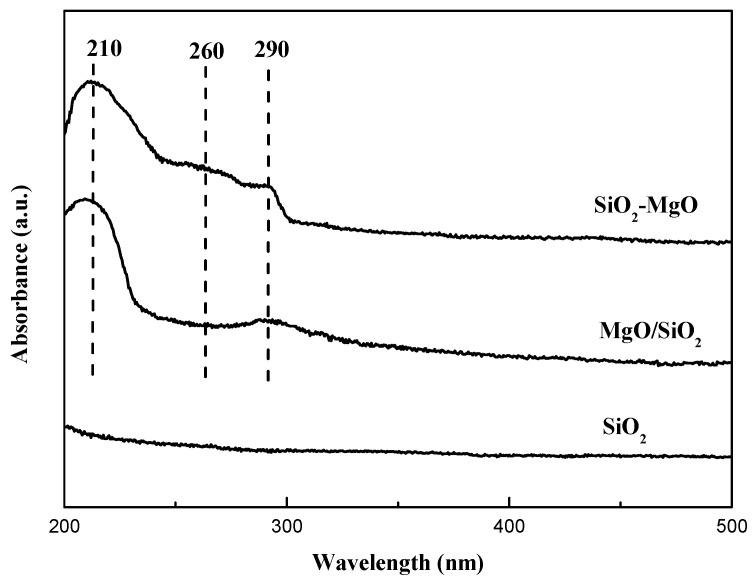
UV-Vis spectra of the supports.

In order to gain further insight into the existence form of Mg species, UV/Vis spectroscopy was performed to characterize the supports. As shown in Figure 4, there were no obvious absorption bands on the spectra of pure SiO_2_. The MgO/SiO_2_ samples show two bands at 213 and 288 nm. According to Coluccia et al. [42,43], for the magnesia–silica samples, the observed absorption bands at 210 and 290 nm were ascribed to the ligand-to-metal charge transfer from four-coordinated (edge or terraces) and three-coordinated (corner) O^2−^ ions to Mg^2+^ of MgO, respectively. The latter absorption band was identified as the more unsaturated and reactive sites on MgO. Base on our results, the two characteristic bands for MgO/SiO_2_ samples are well consistent with the results of the literature. For MgO-SiO_2_ samples, in addition to the above two bands, the absorption band at around 260 nm were observed, which is assigned to Si-O-Mg structures due to the strengthened interaction between MgO and SiO_2_ [44]. Moreover, the 288 nm band of SiO_2_-MgO samples are larger than that of MgO/SiO_2_, suggesting the larger amount of unsaturated sites formed on SiO_2_-MgO samples.

### 3.2. Characterization of the Catalysts

#### 3.2.1. Structure and Texture Properties of the Catalysts

**Figure 5:**
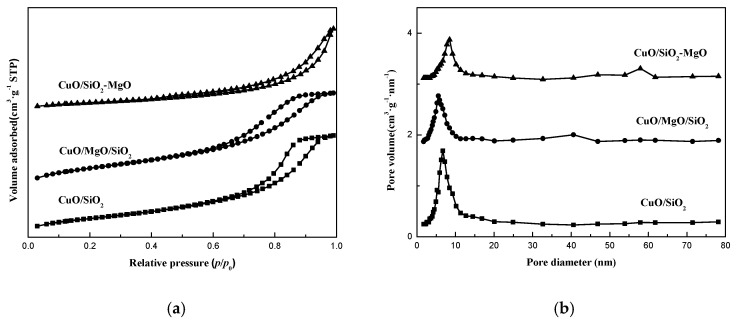
N_2_ adsorption-desorption isotherms (**a**) and pore size distributions (**b**) of catalysts.

As shown in Figure 5, the catalysts showed the similar isotherms as the corresponding supports. Meanwhile, there was no significant difference in the pore size distribution curves compared with the supports, except for the smaller pore diameter and the slightly lower intensity of the peaks. The specific surface area and pore volume of the catalysts are listed in Table 1. The surface area of the catalysts are lower than that of the corresponding supports and in the same descending order with the corresponding supports.

**Figure 6:**
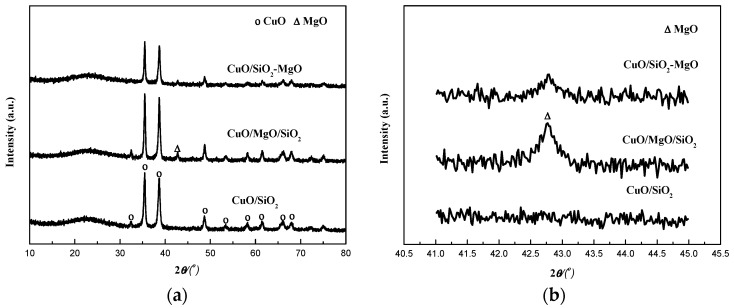
XRD patterns of the catalysts.

The crystalline structures of the catalysts were determined using XRD. As can be seen from the Figure 6a, the featured peak at a *2θ* value of around 22° was attributed to amorphous silica. A series of evident diffraction peaks at 32.6°, 35.5°, 38.7°, 48.8°, 53.4°, 58.3°, 61.7°, 66.2° and 68.1° assigned to CuO (JCPDS 45-0937) were observed in all the catalysts, which partly overlapped the weak and broad peaks of the silica-magnesia supports, especially for the M-S-H species. Compared with CuO/SiO_2_, the higher intensity of the XRD peaks of CuO for CuO/MgO/SiO_2_ catalyst suggested that CuO phase are agglomerated caused by the addition of MgO on the surface of the SiO_2_ support, while the broad and low-intensity peaks of CuO phase for CuO/SiO_2_-MgO catalyst indicated that the employ of SiO_2_-MgO composite support can reduce the CuO particle size and promote its dispersion. Although SiO_2_-MgO support exhibited smallest BET area, the dispersion of CuO in the CuO/SiO_2_-MgO is still higher than that in CuO/SiO_2_ and CuO/MgO/SiO_2_. Moreover, for the three supported catalysts, the copper loadings which were determined by ICP analysis were almost equal (Table 1). According to the above results, it is reasonable to conclude that the dispersion of CuO is mainly dependent on the interaction between the Cu species and the supports other than the simple physical dispersion ability of the supports [32]. The surface new acid-base sites generated from the uneven distribution of charges via the formation of Si-O-Mg structures on the SiO2-MgO might facilitate the dispersion of Cu species, which will be detailedly discussed in the resultes of the surface properties. The crystallite sizes of CuO, calculated by using Scherrer Equation, are listed in Table 1. The CuO crystallite sizes of the CuO/SiO_2_, CuO/MgO/SiO_2_, and CuO/SiO_2_-MgO catalysts were 18.1, 21.3, and 14.7 nm, respectively.

From Figure 6b, the peaks at 42.7°corresponding to the (200) diffraction of MgO can be observed for CuO/MgO/SiO_2_ and CuO/SiO_2_-MgO catalyst, and the crystallinity of MgO followed the same order with that of silica-magnesia supports.

**Figure 7:**
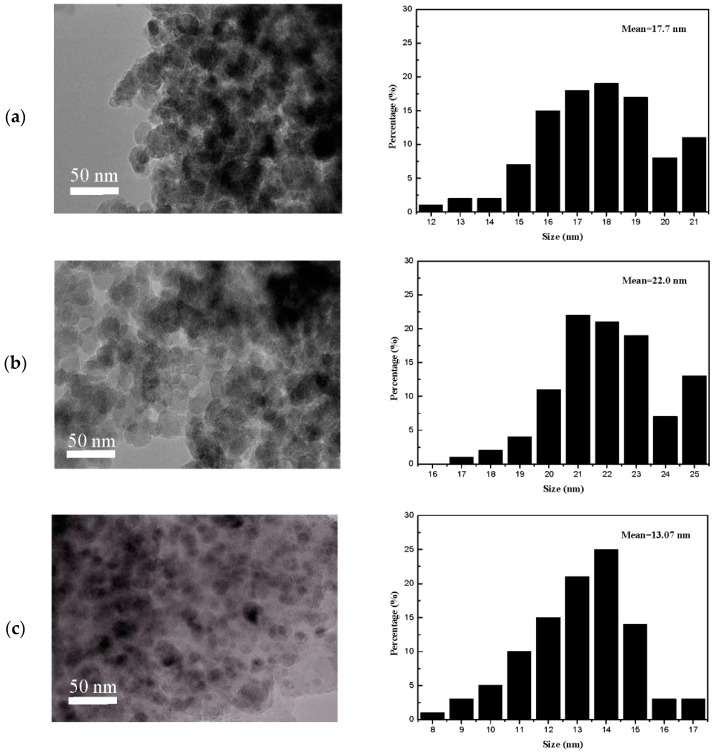
TEM images and particle size distributions of catalysts: (**a**) CuO/SiO_2_; (**b**) CuO/MgO/SiO_2_; (**c**) CuO/SiO_2_-MgO.

The catalysts were also characterized by TEM to further demonstrate the particle size and dispersion. It can be seen from the Figure 7 that the average particle size of CuO was about 17.7 and 22.0 nm for the CuO/SiO_2_ and CuO/MgO/SiO_2_ catalysts, respectively. While, the average particle size of CuO for CuO/SiO_2_-MgO catalyst was about 13.0 nm. The CuO particles were more uniformly disppersed and lessly aggregated on SiO_2_-MgO supports compared with those on SiO_2_ or MgO/SiO_2_ supports, which are in good agreement with the XRD results. It is confirmed again that sintering or aggregation of CuO particles could be effectively prevented owing to the significant interaction of between CuO and SiO_2_-MgO supports.

#### 3.2.2. Surface Properties of the Catalysts

To gain the information about the surface acidity and basicity of the catalysts, NH_3_-TPD, CO_2_-TPD, and Py-FTIR experiments were carried out. 

**Figure 8:**
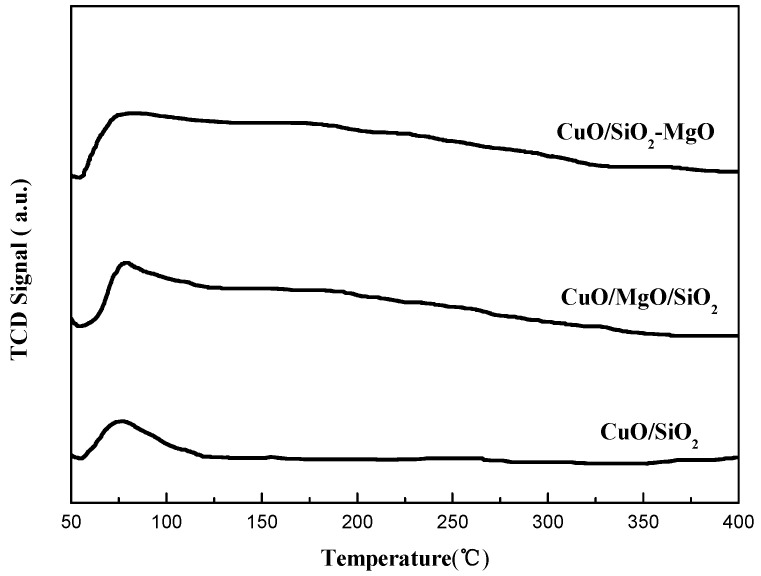
CO_2_-TPD of catalysts.

CO_2_-TPD experiments were performed to examine surface basicity of the catalysts. The TPD profiles contain three main CO_2_ desorption peaks approximately at 90, 165, and 250 °C, which can be attributed to the weak (OH-groups), moderate (Mg-O-Mg groups), and strong basic surface sites (coordinatively unsaturated O^2−^ ions) [26,27,28]. As shown in Figure 8, only one broad peak was obtained at 90 °C for CuO/SiO_2_ samples, corresponding to the weak basic sites. However, the different peaks higher than 90 °C generated in CuO/SiO_2_-MgO and CuO/MgO/SiO_2_ catalysts indicated that the proportion of medium and strong basic sites on the surface varied with the existence of Mg species. Moreover, the basicity of the catalysts calculated from the CO_2_-TPD peak areas are also summarized in Table S1. The number of medium-strong basic sites varies in the order: CuO/SiO_2_-MgO > CuO/MgO/SiO_2_ > CuO/SiO_2_. The CuO/SiO_2_-MgO catalysts showed a greater basicity than the others in terms of basic sites and strength, which may be caused by two main reasons. The one could be due to the highly dispersed MgO microcrystals confirmed by the XRD, which may expose more O_LC_^2−^ (LC = low coordination) sites at terraces, edges and corners [27]. On the other hand, the bridged O with enhanced electron cloud density in Si-O-Mg structure could also contribute to the surface basity [35].

**Figure 9:**
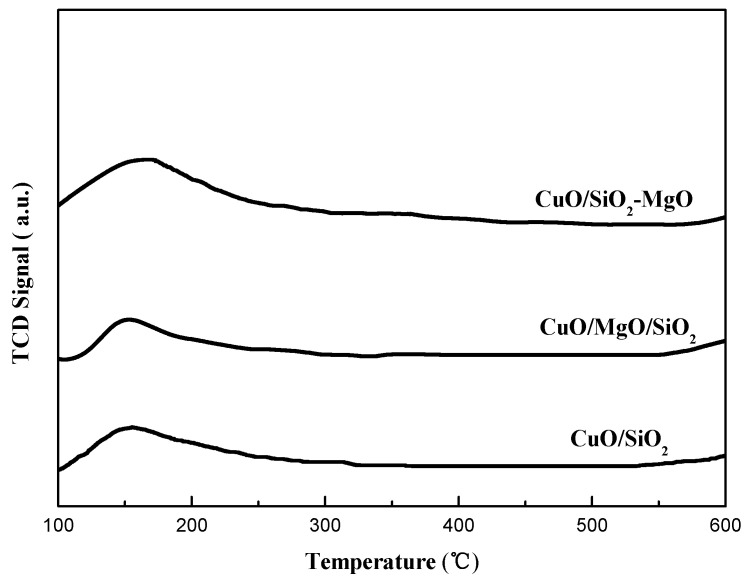
NH_3_-TPD of catalysts.

The NH_3_-TPD profiles (Figure 9) of all the catalysts showed one low-temperature peak at 170–200 °C, which was ascribed to NH_3_ desorpted from weak acid sites. For CuO/SiO_2_ catalysts, as reported earlier, these weak acid sites derived from the coordination unsaturated CuO [45]. Obviously, compared with CuO/SiO_2_ catalysts, the introduction of Mg species did not have significant effect on the surface acidity of the catalysts. However, according to the previous studies, both of the doping of MgO species in to Cu/SiO_2_ catalysts and the formation of Si-O-Mg structure could give rise to the increasement of the surface acidity of the catalysts [35,46]. At the present study, the reason for no obvious changes on the acidity of the catalysts may be due to the combined effects of the neutralizing by the surface basic sites and the covering by the copper species [46].

**Figure 10:**
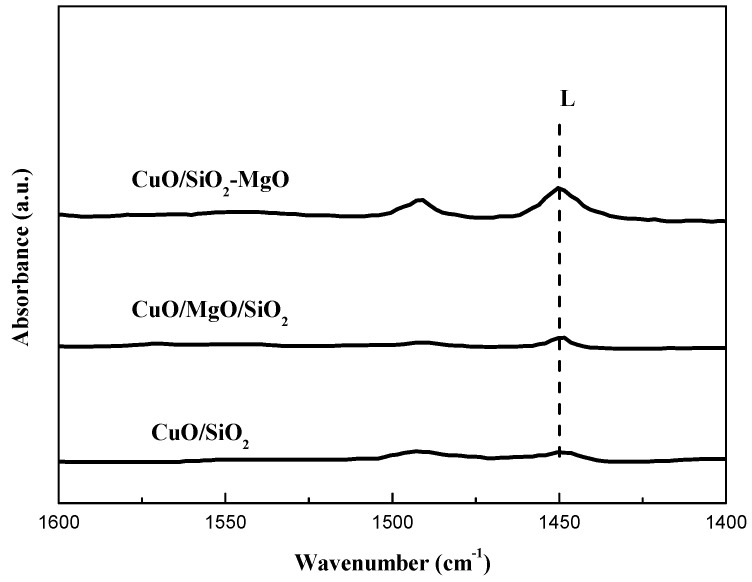
Py-IR spectra of catalysts.

**Figure 11:**
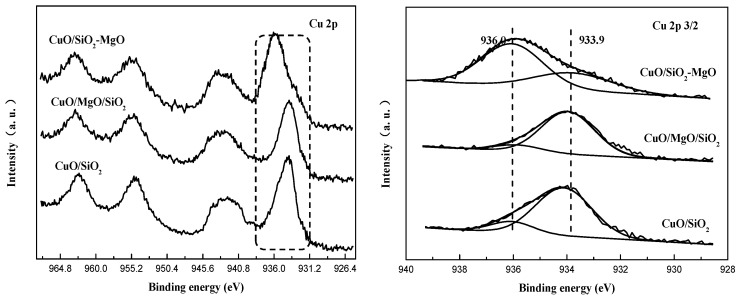
Cu 2p XPS spectra of the catalysts.

Figure 10 illustrates the pyridine infared spectra (Py-FTIR) of catalysts in the range of 1400–1700 cm^−1^. According to the literature [47]: the IR bands around 1540 and 1630 cm^−1^ can be ascribed to pyridinium ions on Brönsted acid sites, while the bands at 1450 and 1610 cm^−1^ can be attributed to pyridine molecules coordinated to Lewis acid sites. In addition, the band around 1490 cm^−1^ can be assigned to the contribution of pyridine interacting with both Lewis and Brönsted acid sites. As can be seen from the Figure 11, all catalysts exhibit Lewis acid sites but the band of Brönsted acid at 1540 cm^−1^ was not found in the IR-spectra. We also presents the Py-IR spectra of the corresponding supports for comparison in Figure S1. The acid type on the SiO_2_ and MgO/SiO_2_ supports is similar to the corresponding catalysts. While, for the SiO_2_-MgO support, not only the Lewis acid sites but also the Brönsted acid sites were observed. The imbalanced charge caused by the substitution of Si^4+^ by Mg^2+^ results in the protons remaining on the SiO_2_-MgO support which interacted with O^2^^−^ ions to form the Brönsted acid sites [48]. For the CuO/SiO_2_-MgO samples, the vanishment of the Brönsted acid sites may be due to the replacement of the hydroxyl protons on the SiO_2_-MgO support surface by the copper species during the preparation of the catalysts, which is similar to the ion exchange between Cu^2+^ and H^+^ in the hydroxyl groups (Si-O-Mg) on the support and results in the Cu-O-Si units [49,50]. The formation of Cu-O-Si units indicates the strong interaction between copper species and the SiO_2_-MgO support [13].

#### 3.2.3. Identifying the Cu-Support Interaction

XPS were carried out to determine the chemical environment of Cu species (Figure 11). For all the catalysts, the intense and broad Cu 2p_3/2_ peak together with an obvious shakeup satellite peak locate at around 933.9 eV at and 942.0 eV, repectively, suggesting the only existence of Cu^2+^ species [51,52]. The asymmetric Cu 2p_3/2_ peaks of all the catalysts can be fitted into two peaks centered at 936.0 eV and 933.9 eV, indicating there were two distinct types of Cu^2+^ species in the supported catalysts. Cu^2+^ species centered at 933.9 eV are due to the bulk CuO and the higer BE ones at 936.0 eV are attributed to the well-dispersed Cu^2+^ ions interacting with the support [46,53]. It can be seen from the change of the fitting peaks of Cu 2p_3/2_ that for CuO/SiO_2_-MgO samples, Cu^2+^ species at 936.0 eV reach the maximum and the main body of the Cu 2p_3/2_ peak shifted to the higher binding energy, indicating the stronger interaction between copper species and the support.

**Figure 12:**
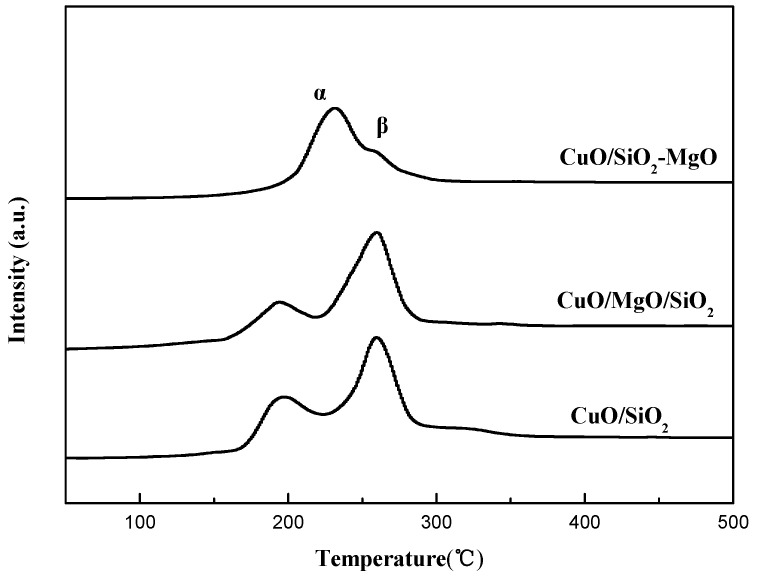
H_2_-TPR profiles of the catalysts.

H_2_-TPR of the samples was studied and the profiles are displayed in Figure 12. Due to the high reduction potential (E°-2.372 V) of MgO which makes it hard to be reduced, the total hydrogen consumption is due to the reduction of the CuO. All samples exhibited two reduction peaks α (around 200 °C) and β (around 250 °C) indicating the existence of two kinds of CuO species, which is consistent with the results of XPS. It is considered that the α peak is due to the small Cu^2+^ species interacted with supports, and the β peak is ascribed to the reduction of the larger CuO particles [46,53]. The area ration of peaks α and β followed the sequence of CuO/SiO_2_-MgO > CuO/SiO_2_ > CuO/MgO/SiO_2_, suggesting the size decrease and better dispersion of the CuO particles, which is well consistent with the XRD and TEM results. The interaction between the Cu species and the supports can effectively affects its reduction behaviors. It’s worth noting that, among these catalysts, the α peak of CuO/SiO_2_-MgO shifted to higher temperature compared by CuO/SiO_2_ and CuO/MgO/SiO_2_. The primary reason for this phenomenon is that the well dispersed Cu^2+^ species generated from the Cu-O-Si units possess the lower reduction capacity [13], and then result in the increase of the α peak temperature. On account of the pronounced interaction, the copper species in CuO/SiO_2_-MgO dispersed well, and thus the grain size was smaller than that of CuO/MgO/SiO_2_ with weak Cu-support interaction. The crystalline size of copper species in CuO/SiO_2_ was between them, as well as the interaction.

### 3.3. Catalytic Performance

For the ethynylation reaction, the main reaction process can be written as follows:

HC≡CH + 2HCHO → HO-CH_2_C≡CCH_2_-OH

**Figure 13:**
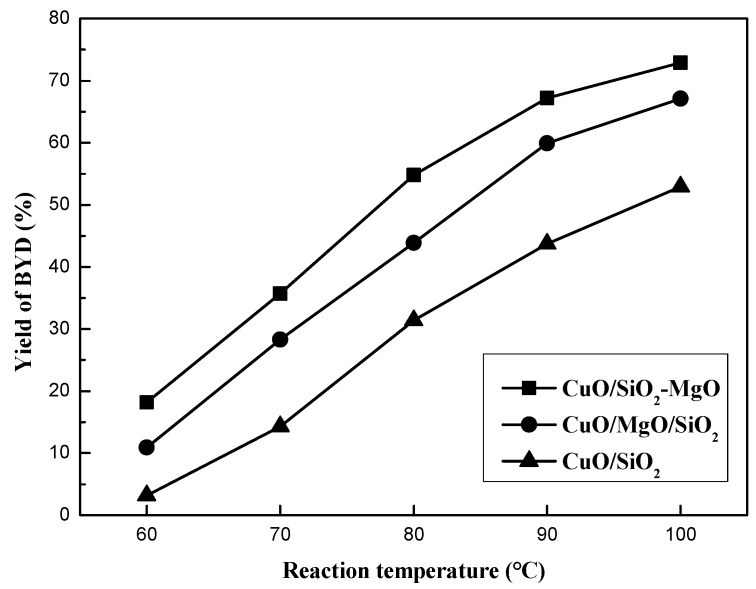
Effect of reaction temperature on the yield of BD.

Figure 13 displays the catalytic performance of all the catalysts with different reaction temperatures under atmospheric pressure in 15 h. BD was the only quantifed carbon-containing products, and no other byproducts such as Propargyl alcohol, Formic acid and polyethyne were quantifed because of their very low content. Reaction temperature was found to have a great influence on the catalys performance. The yield of BD can be greatly improved with the temperature elevated. Overall, ternary catalysts exhibited better catalytic performance than CuO/SiO_2_, indicating that the introduction of the Mg species into the CuO/SiO_2_ catalyst significantly improved its catalytic activity. Neverthless, CuO/SiO_2_-MgO catalyst exhibited the better BD yield than that of CuO/SiO_2_/MgO catalyst. Table S2 lists the catalytic activity of the three catalysts and their supports under the reaction temperature of 90 °C. The yield of BD over CuO/SiO_2_ was only 35.4%, while that of CuO/MgO/SiO_2_ and CuO/SiO_2_-MgO was 56.5% and 67.2%, respectively. Thereinto, CuO/SiO_2_-MgO showed the highest yield of BD. These results suggesed that the ethynylation activity of the catalysts could be improved in varying degrees via the different existential states of the Mg species.

**Figure 14:**
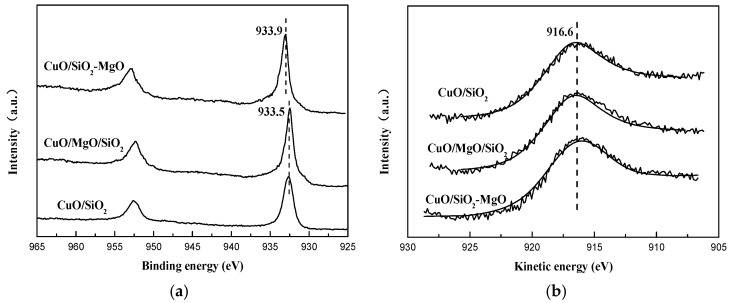
XPS spectra (**a**) and LMM XAES spectra (**b**) of the catalysts after reaction after reaction.

It is generally acknowledged that the cuprous acetylide is the real active sites [11,12]. The process of CuO phase transformation during the reaction will cause the change of the valence state of the copper species. However, to the best of our knowledge, there is no direct evidence to prove the valence state of the active copper species. Thus, XPS and XAES are carried out to determine the valence states of the Cu species on the surface of the used catalyst. As shown in Figure 14a, compared with the fresh catalysts, the BE value of Cu 2p_3/2_ shifts to around 932.5 ev and the satellite peak disappears. This phenomenon is due to the reduction of Cu^2+^ to Cu^+^ and/or Cu^0^ [32]. The BE value of Cu 2p_3/2_ in CuO/SiO_2_-MgO is higher than the other two catalysts, indicating the strong interaction between copper species and the SiO_2_-MgO support still exits even after the evolution of the phase of the copper species [54]. It is well know that the the positive shift of BE suggesting a charge transfer from the metal ions toward the support, which results in the lower the outer shell electronic density in the used samples. This results may be the reason why the Cu^2+^ interacted with the SiO_2_-MgO support is much more difficult to be reduced under identical conditions [54]. Due to the similar BE value between Cu^+^ and Cu^0^ species, it is hard to distinguish the copper species with low valence via using XPS. Therefore, the Cu LMM XAES spectrum is employed to discriminate the copper species. As displayed in Figure 14b, the symmetric Auger kinetic energy peak at around 916.6 eV corresponding to Cu^+^ species could be observed [13,32], indicating only Cu^+^ species exits on the catalysts.

**Figure 15:**
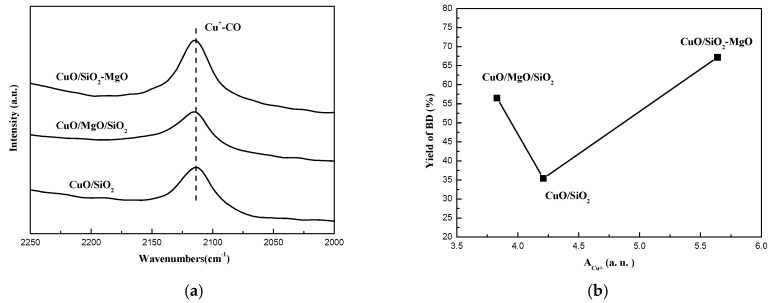
CO-IR spectra of catalysts after reaction (**a**) and Relationship between BD yield and A_Cu_+ (**b**).

CO-IR is an appropriate measure for characterization of Cu^+^ sites, where molecular CO can be strongly adsorbed to form carbonyls. As presented in Figure 15a, only one peak at 2120 cm^−1^ was observed in the IR-spectra of each catalyst, which was ascribed to the linear adsorption of CO molecules on the Cu^+^ sites according to the Cu^n+^-CO system detailed by the previous literature [55,56,57,58,59]. Moreover, according to the semi-quantity method for the amounts of Cu^1+^ by Xue et al. [60], we calculated the integration area of the Cu^1+^-CO band and the results are listed in Table 1. The amount of Cu^+^ follow the order as CuO/SiO_2_-MgO > CuO/SiO_2_ > CuO/MgO/SiO_2_, which is consistent with the dispersion of CuO on the various supports. Figure 15b displayed the relationship between the amount of Cu^+^ species of each catalysts and the corresponding BD yield. With the increasing amounts of Cu^+^ species, the activity first decreased and then increased. In general, the high amount of Cu^+^ exposed on the catalyst surface were favorable for the ethynylation reaction [11,12]. However, Cu^+^ species was minimal on the Cu/MgO/SiO_2_ catalysts, the catalytic activity over Cu/MgO/SiO_2_ catalysts is much higher than Cu/SiO_2_. This results triggered us to consider the other factors that affect the activity of the catalysts, which will be discussed in the next section.

**Figure 16:**
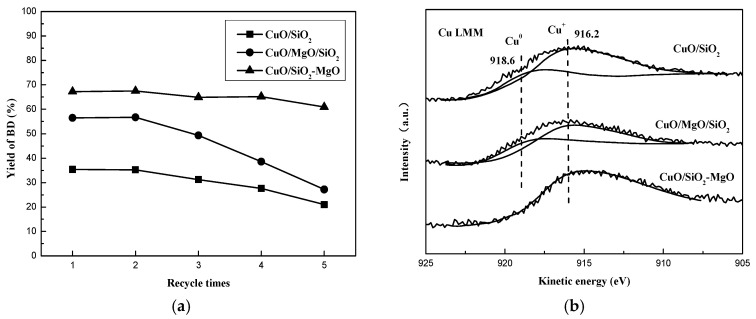
Cyclic test of catalysts (**a**) and Cu LMM XAES spectra of catalysts after 5 cycles (**b**).

Stability of the ethynylation catalysts is a critical index for both the practical industrial and academic viewpoints, thus the 5 cycles of evaluation over the prepared catalyts was investigated. As shown in Figure 16a, no apparent decrease in the BD yield was obverved over CuO/SiO_2_-MgO, while the obvious deactivation was found over CuO/MgO/SiO_2_ (59.9% to 24.9%) as well as CuO/SiO2 (43.7% to 22.3%). CuO/SiO_2_-MgO exhibited remarkable stability in compared with the others. The appearance of metallic Cu^0^ leading to the loss of active sites is considered as the predominant factor for catalyst deactivation in the ethynylation reaction [13]. Thus, the Cu LMM XAES of the catalysts after 5 cycles were performed to identify the change of the copper species valence. As displayed in Figure 16b, for the CuO/MgO/SiO_2_ and CuO/SiO_2_ catalysts, the broad and asymmetric Auger kinetic energy peaks could be fitted into the peaks of Cu^+^ and Cu^0^ species, which centered at around 916 eV and 918 eV, respectively [13]. However, there was only one Auger kinetic energy peak over CuO/SiO_2_-MgO catalyst, indicating that after 5 cycles of evaluation, the surface copper speices of CuO/SiO_2_-MgO catalyst is sill dominated by Cu^+^ species. The quantitative results of the Cu^+^/(Cu^+^ + Cu^0^) are summerized in Table 1. It can be seen that this value gets the almost 100.0% on the CuO/SiO_2_-MgO catalyst. Additionally, for the CuO/MgO/SiO_2_ and CuO/SiO_2_ catalysts, the values are 81.7% and 82.1%, respectively. This can be considered as a benificial evidence to demonstrate that the strong interaction with SiO_2_-MgO support can alter the reduction behavior of the copper species and stablize more active sites (Cu^+^) during the catalytic reaction. On the basis of the above results, it can be found that the formation of metallic Cu is in good agreement with the decrease of the catalysts stability. Interestingly, the declining proportions of Cu^+^ on the CuO/MgO/SiO_2_ catalyst is very close to that on the CuO/SiO_2_ catalysts, while the activity of CuO/MgO/SiO_2_ catalyst dropped even faster.

## 4. Discussion

In this study, we tried to introduce different existential states of Mg species into the CuO/SiO_2_ catalysts. It is found that the existential states of Mg species influence not only the physichemical properties of silica-magnesia composite oxides but, more importantly, the existence state of CuO and surface properties of the corresponding supported catalysts. Based on the XRD, IR, UV-Vis and N_2_-physisorption results of the supports, the physichemical state of Mg species are different between MgO-SiO_2_ and SiO_2_-MgO samples. It is reasonably to conclude the MgO microcrysals and Si-O-Mg structure co-exist in SiO_2_-MgO samples, while there were only MgO particles on the MgO/SiO_2_ samples. Py-IR results of the supports demonstrated that Brönsted acid sites were formed on the SiO_2_-MgO supports. The Brönsted acid sites may be due to the formation of Si-O-Mg hetero-linkages in binary complex oxides which can generate the uneven distribution of charges and protons will stay on the surface to ballance the uneven distribution of charges. In fact, these protons interacte with O anions to form hydroxyls. Similar interpretaion of the Brönsted acid sites models formed on the SiO_2_-Al_2_O_3_ were proposed by Thomas [48]. Moreover, the disparity of Mg species generated from preparation methods also show different effects on the texture of supports.

The surface area and crystalline structure of the synthesized supported catalysts are greatly changed by the different supports. For these three supported catalyst, the weak Cu-support interaction are the principal reason for the poor dispersion of copper species. In spite of the biggest S_BET_ of CuO/SiO_2_, the CuO dispersion of CuO/SiO_2_ was worse than that of CuO/MgO-SiO_2_. In contrast, CuO/MgO-SiO_2_ showed the lowest S_BET_, the dispersion of copper species was the best among the catalysts, indicating that an interaction exceeded simple physical dispersion ability exists between Cu species and MgO-SiO_2_. From the H_2_-TPR results, two kinds of copper species were observed. For CuO/MgO-SiO_2_ catalyst, the αpeak shifted to higher temperature and the peak area increased, indicating the strong interaction between between copper species and SiO_2_-MgO support. The XPS results also confirmed the observations in H_2_-TPR. Py-IR results showed that, compared with MgO-SiO_2_ support, the Brönsted acid sites on the CuO/MgO-SiO_2_ catalyst disappeared. The similar phenomenon was reported by Meng et al. [50]. They found that copper ions could interact with H sites in the hydroxyl groups of the TiO_2_-SiO_2_ xerogel, which enhances the Cu-support interaction. According to the difference in TPR, XPS, and Py-IR spectra, we could steadily conclude that the strength of the interaction between copper species and support exists in the catalysts following the sequence of CuO/MgO-SiO_2_ > CuO/SiO_2_ > CuO/MgO/SiO_2_.

In ethynylation reaction, it is widely accepted that cuprous species (cuprous acetylides) act as the main active sites. Trotuş et al. [3] also pointed that it is actually the cuprous acetylides formed during the reaction that catalyzes the reaction, suggesting the valence of the copper species will change from Cu^2+^ to Cu^1+^. This view was directly confirmed by the results of the XPS spectra and the X-ray induced Auger spectra (XAES) of the used catalysts. The amount of Cu^+^ on the catalyst surface is ordered: Cu/MgO-SiO_2_ > Cu/SiO_2_ > Cu/MgO/SiO_2_, which is also consistent with the sequence of the strength of Cu-support interaction. Previous study have shown that the strong interaction between copper species and support could retard the surface transmigration of copper species and greatly improve the dispersion of the copper species [11,32]. Meanwhile, the strong interaction could alter the reduction behavior of CuO, which could stablize the valence of Cu^+^ [13]. The stability test of the catalysts and the corresponding Cu LMM XAES characterization results further confirmed the above viewpoints.

Highly dispersed and valence-stable cuprous species are responsible for the better activity in ethynylation of formaldehyde [11,12]. We correlated the integration area of CO-IR with the catalytic activity (yield of BD in 15 h). As can be seen from the Figure 15b, Such a reverse volcano trend with the amounts of Cu^+^ species indicats that in addition to the amounts of the surface Cu^+^ species, there might be other factor affecting the catalytic activity. Based on the characteristics of the reactant molecules, namely the terminal ≡C-H of the acetylene molecule and the C=O unit of the formaldehyde molecule, we further attempted to associate the surface acidity/basicity of the catalysts with their catalytic activity.

**Figure 17:**
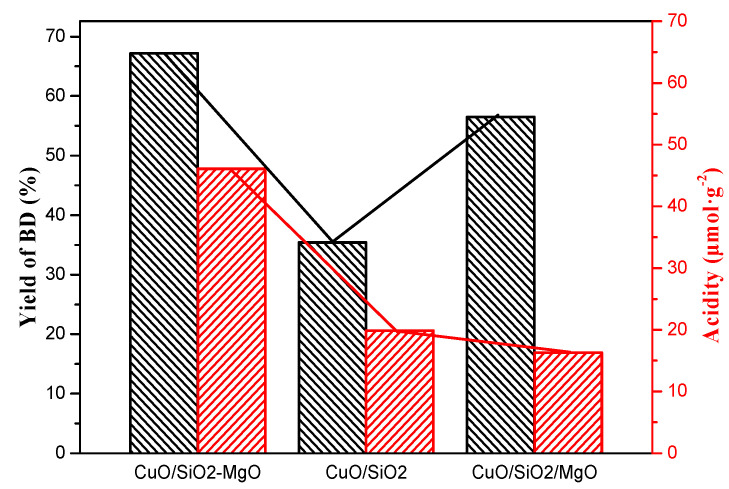
Relationship between BD yield and Lewis acid sites.

According to the study of Li et al. [61], Lewis acid sites could activate the carbonyl and enhance activity of enthynylation. Therefore, the yield of BD might be related to the surface Lewis acidity of the catalysts. As shown in Figure 17, the variation trend of BD yield deviates from that of surface acidity. From the NH_3_-TPD and Py-IR results of the catalysts, the introduction of Mg species has little effect on the acid strength of the catalysts, and the catalysts exhibit weak acidity. For the silica-magnesia supports with a very large band gap, the surface Lewis acid sites mainly originated from the Si or Mg ions, which are less oxophilic than Zn, Ir, Zr, and Ru ions etc. [62]. Therefore, there are few report about the activation of C=O group by Si or Mg ions. Combined the above reasons, we consider that the surface acidity of the catalysts is not intrinsic factor for affecting the BD yield.

**Figure 18:**
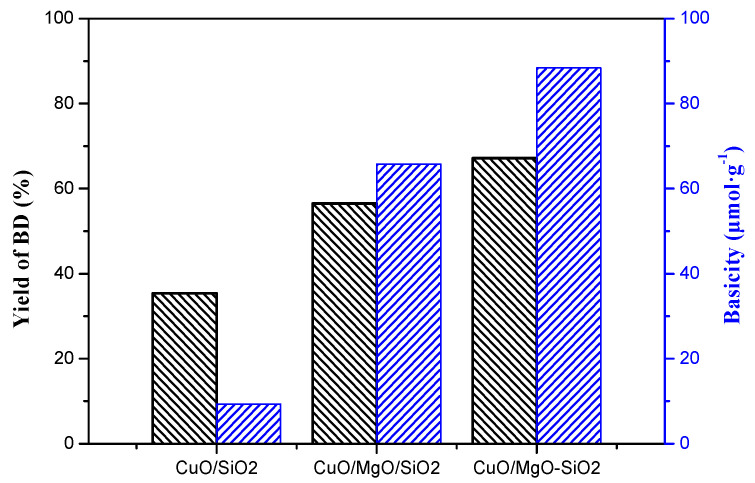
Relationship between BD yield and medium-strong basic sites.

On the other hand, the introduction of Mg species bring about a large number of basic sites, especially medium and strong basic sites. Figure 18 obviously presented that the yield of BD increased with an increasing amount of medium-strong basic sites of the catalyst. As many studies reported [63,64], bases are regarded as an important reason in favor of catalystic enthynylation performance. The basic sites can bind to an acidic proton in acetylene, which enhances the nucleophilicity of the acetylenic carbon. A strongly nucleophilic acetylenic carbon (≡C-) is favorable for attack at the electropositive C in the C=O unit of molecular formaldehyde, which promotes the formation of BD. Remarkably, CuO/SiO_2_-MgO catalyst possessed the maximum active Cu^+^ sites and basic sites, showing a strongly synergistic effect and the best catalytic activity. In addition, the rapid decrease of the CuO/MgO/SiO_2_ catalysts during the 5 cycles of evaluation is probably due to the imbalance between the Cu^+^ species and the basic sites caused by the reduction of the partial Cu^+^ species to the inactive Cu^0^. This phenomenon further clearly indicates that the basicity of the catalysts played a key role in the enthynylation reaction.

## 5. Conclusions

In this study, the CuO/SiO_2_ catalysts were modified with Mg species of different existential states, then employed for the ethynylation of formaldehyde reaction. The existence form of Mg species on the catalysts influenced the copper–support interaction, the surface basicity and the ethynylation performance. Based on the present study, we can draw the conclusions as follows:

(1) The strong interaction derived from the formation of Si-O-Mg structures in SiO_2_-MgO support conduced to altering the dispersion and the reduction behavior of the copper species. The highly dispersed and stablized Cu^+^ species formed on the SiO_2_-MgO support are responsible for the superior catalytic stability.
(2) The incorporation of Mg species also altered the basic property on the surface of the catalysts. In this catalytic system, the medium-strong basic sites play a key role in ≡C-H activation due to their deprotonation ability, showing a strongly synergistic effect with Cu^+^ species, resulting in a dramatical improvement in ethynylation activity.

Our work elucidates an understanding of the promotional roles of different Mg species in the structure and ethynylation performance of Cu/SiO_2_ catalysts, which is considerable interest in term of the Reppe process and provides guidance for the design of new catalyst system.

## Figures and Tables

**Table 1:** Structural and textural data for the CuO-Bi_2_O_3_ catalysts.

Samples	Cu Content (wt.%) ^a^	S_BET_ (m^2^·g^−1^) ^b^	V_p_ (cm^3^·g^−1^) ^b^	d_CuO_ (nm) ^c^	Cu^+^/(Cu^0^+Cu^1+^) (%) ^d^	A_Cu_+ ^e^ (a.u.)
SiO_2_	-	1031.7	2.31	-	-	-
MgO/SiO_2_	-	898.5	2.11	-	-	-
SiO_2_-MgO	-	753.6	1.97	-	-	-
CuO/SiO_2_	16.7	642.7	1.01	18.1	82.1	4.2
CuO/MgO/SiO_2_	17.5	499.2	0.92	21.3	81.7	3.8
CuO/SiO_2_-MgO	17.3	385.8	0.88	14.7	100	5.6

Note: ^a^ Cu bulk loading was determined by ICP-AES analysis; ^b^ S_BET_ was calculated by the BET method; ^c^ d_CuO_ was calculated from the reflections of CuO (111) plane in the XRD using the Scherrer equation; ^d^ Cu^+^/(Cu^0^ + Cu^1+^) was calculated from the Cu XAES spectra of the catalysts after 5 cycles; ^e^ A_Cu_+ was obtained by the integration of IR bands areas of Cu^+1^-CO.

## Supplementary Materials

The following are available online at https://www.mdpi.com/2079-4991/9/8/1137/s1, Figure S1: Py-IR spectra of supports. Figure S2: NH_3_-TPD spectra of supports. Figure S3: XPS spectra of supports. Table S1: Acid-base properties of supports and catalysts. Table S2: Ethynylation performace of catalysts under the reaction temperature of 90 °C.
