# Correction: Wang et al. Ethynylation of Formaldehyde over CuO/SiO_2_ Catalysts Modified by Mg Species: Effects of the Existential States of Mg Species. *Nanomaterials* 2019, *9*, 1137

**DOI:** 10.3390/nano15080599

**Published:** 2025-04-14

**Authors:** Zhipeng Wang, Lijun Ban, Pingfan Meng, Haitao Li, Yongxiang Zhao

**Affiliations:** Engineering Research Center of Ministry of Education for Fine Chemicals, School of Chemistry and Chemical Engineering, Shanxi University, Taiyuan 030006, China

In the original publication [1], there was a mistake in Figure 1 as published. Authors corrected the XRD data of SiO_2_ and re-made Figure 1. The corrected Figure 1 appears below. The authors state that the scientific conclusions are unaffected. This correction was approved by the Academic Editor. The original publication has also been updated.

## Figures and Tables

**Figure 1:**
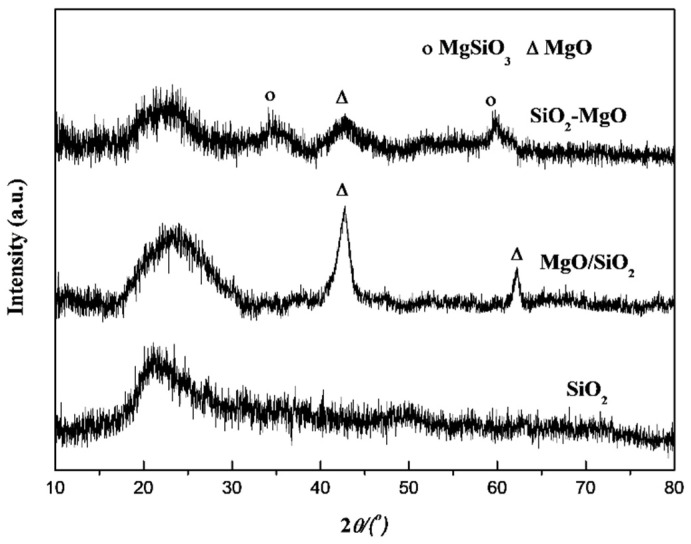
XRD patterns of the supports.
